# W-IQ-TREE: a fast online phylogenetic tool for maximum likelihood analysis

**DOI:** 10.1093/nar/gkw256

**Published:** 2016-04-15

**Authors:** Jana Trifinopoulos, Lam-Tung Nguyen, Arndt von Haeseler, Bui Quang Minh

**Affiliations:** 1 Center for Integrative Bioinformatics, Max F. Perutz Laboratories, University of Vienna, Medical University of Vienna, 1030 Vienna, Austria; 2 Bioinformatics and Computational Biology, Faculty of Computer Science, University of Vienna, 1090 Vienna, Austria

## Abstract

This article presents W-IQ-TREE, an intuitive and user-friendly web interface and server for IQ-TREE, an efficient phylogenetic software for maximum likelihood analysis. W-IQ-TREE supports multiple sequence types (DNA, protein, codon, binary and morphology) in common alignment formats and a wide range of evolutionary models including mixture and partition models. W-IQ-TREE performs fast model selection, partition scheme finding, efficient tree reconstruction, ultrafast bootstrapping, branch tests, and tree topology tests. All computations are conducted on a dedicated computer cluster and the users receive the results via URL or email. W-IQ-TREE is available at http://iqtree.cibiv.univie.ac.at. It is free and open to all users and there is no login requirement.

## INTRODUCTION

IQ-TREE (1), the successor of the TREE-PUZZLE program (2), is an efficient and versatile phylogenetic software for maximum likelihood analysis of large phylogenetic data. IQ-TREE explores the tree space efficiently and often achieves higher likelihoods than RAxML (3) and PhyML (4). Other key features of IQ-TREE are (i) very fast model selection procedure including partition scheme finding (5), (ii) partitioned analysis for phylogenomic data (6), (iii) ultrafast bootstrap approximation (7), and (iv) implementation of several branch tests (8) and (v) tree topology tests (e.g. (9)).

Most phylogenetic software packages (including IQ-TREE) are command line based, and therefore laborious to run for non-experts. Thus, many web applications with intuitive user-interface were developed (e.g. (10,^11^)).

**Figure 1.**
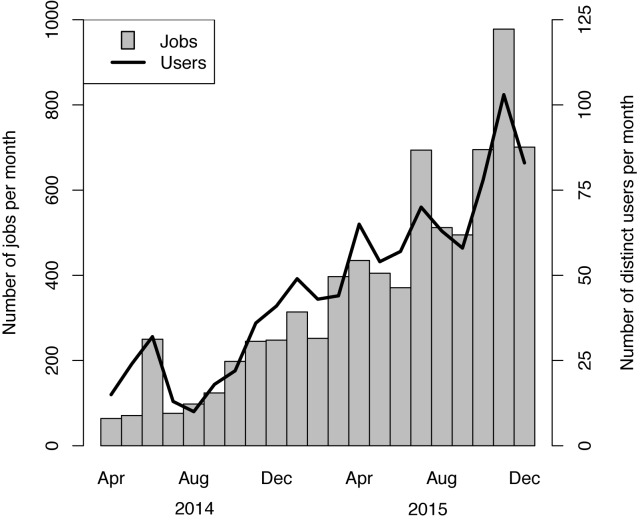
Number of all W-IQ-TREE jobs per month irrespective of the IP-addresses submitted by external users and number of distinct users per month.

Here, we present W-IQ-TREE, a user-friendly web application and compute server for phylogenetic analyses with the IQ-TREE software. W-IQ-TREE currently runs on a computer cluster with 32 CPUs, which can be extended depending on the usage. Since its first launch in April 2014 the numbers of users and submitted jobs are steadily increasing (Figure 1). This is most likely attributed to the user-friendly features presented below.

## IQ-TREE WEB APPLICATION

W-IQ-TREE was designed to work on all web browsers. It provides a web interface to interact with users and send user requests to the computer cluster, where the actual computation is done with the most recent sequential IQ-TREE version. In the following, we describe important elements of the web interface.

### Input data

W-IQ-TREE accepts input alignments in PHYLIP, FASTA, Nexus, Clustal or MSF format. Various sequence data are supported: DNA, amino acids, codons, binary and morphological data. Binary sequences are encoded by 0 and 1 whereas morphological sequences allow 0–9 and A–Z as characters. For phylogenomic alignments, users can supply a partition file defining a partitioning scheme, for example, to specify different genes or to distinguish between codon positions.

### Models of sequence evolution

By default, W-IQ-TREE will determine the best-fit substitution model (see below) followed by tree reconstruction. Alternatively, users can specify the substitution model together with models of rate heterogeneity like the discrete Gamma (12) and the FreeRate model (13). IQ-TREE supports a wide range of substitution models including protein mixture models (14,15). An ascertainment bias correction model (16,17) can also be switched on to correct the likelihoods if the alignment does not contain invariable sites (e.g., single nucleotide polymorphism or morphological data).

### Model selection

W-IQ-TREE supports a ‘standard’ model selection procedure like jModelTest (18) and ProtTest (19) as well as an extended procedure (i.e. including the FreeRate heterogeneity model). The FreeRate heterogeneity model relaxes the discrete Gamma model by ‘freely’ estimating rates and proportions of the site categories. W-IQ-TREE uses the Bayesian information criterion (20) (default) or the Akaike information criterion (21) to select the best-fit model. For phylogenomic data, W-IQ-TREE determines the best-fit partitioning scheme using a fast implementation of PartitionFinder (5).

### IQ-TREE search parameters

IQ-TREE implements a stochastic algorithm to sample local optima in the tree space. To this end, IQ-TREE maintains a set of candidate trees and applies an evolutionary search algorithm to improve the candidate set. This procedure iteratively performs two operations: perturbing a candidate tree and locally optimizing the perturbed tree by nearest neighbor interchange (NNI). They are controlled by two search parameters: $p$, the perturbation strength, and $c$, the number of iterations since the last best tree was found.

In the default setting, $p$ is set to 0.5 (i.e. half of the internal branches are randomly perturbed by NNI) and $c$ equals 100 (i.e. IQ-TREE stops if no better tree was found within the last 100 iterations). Although this setting was empirically determined to work well (1), it might not hold true for all data sets. For data sets with many sequences, users should specify a higher $c$ to explore the tree space more extensively. For short sequences a smaller $p$ is recommended, whereas for long sequences a larger $p$ allows for broader sampling of the tree space. It is also recommended conducting multiple IQ-TREE runs using different search parameters.

### Branch support analysis

W-IQ-TREE provides a number of methods to assess the reliability of internal branches: standard bootstrap (22), the SH-aLRT (4), aBayes test (8) and the ultrafast bootstrap (7) (UFBoot).These tests can be combined in a single run. The UFBoot has two parameters that can be set via the web interface: the minimum correlation coefficient (default: 0.99) and the maximum number of iterations (default: 1000). Here, UFBoot computes the Pearson correlation coefficient of two sets of support values during the analysis. UFBoot stops as soon as the maximum number of iterations is reached or if the correlation between the two sets of support values exceeds 0.99, which works for most data sets. When the alignment contains little phylogenetic information, the correlation between the two sets of support values might not exceed 0.99. In such a case, users are advised to increase the maximum number of iterations.

### Tree topology evaluation and tests

If users provide a tree file containing several trees in NEWICK format, W-IQ-TREE will compute the log-likelihoods for all given trees. Here, IQ-TREE estimates model parameters (e.g. substitution rates) on a parsimony tree and only optimizes the branch lengths of the user trees to save computation. Moreover, W-IQ-TREE performs several tree topology tests including the KH test (23), the SH test (24), the approximately unbiased (AU) test (9) and the expected likelihood weight (25).

### Analysis results

**Figure 2.**
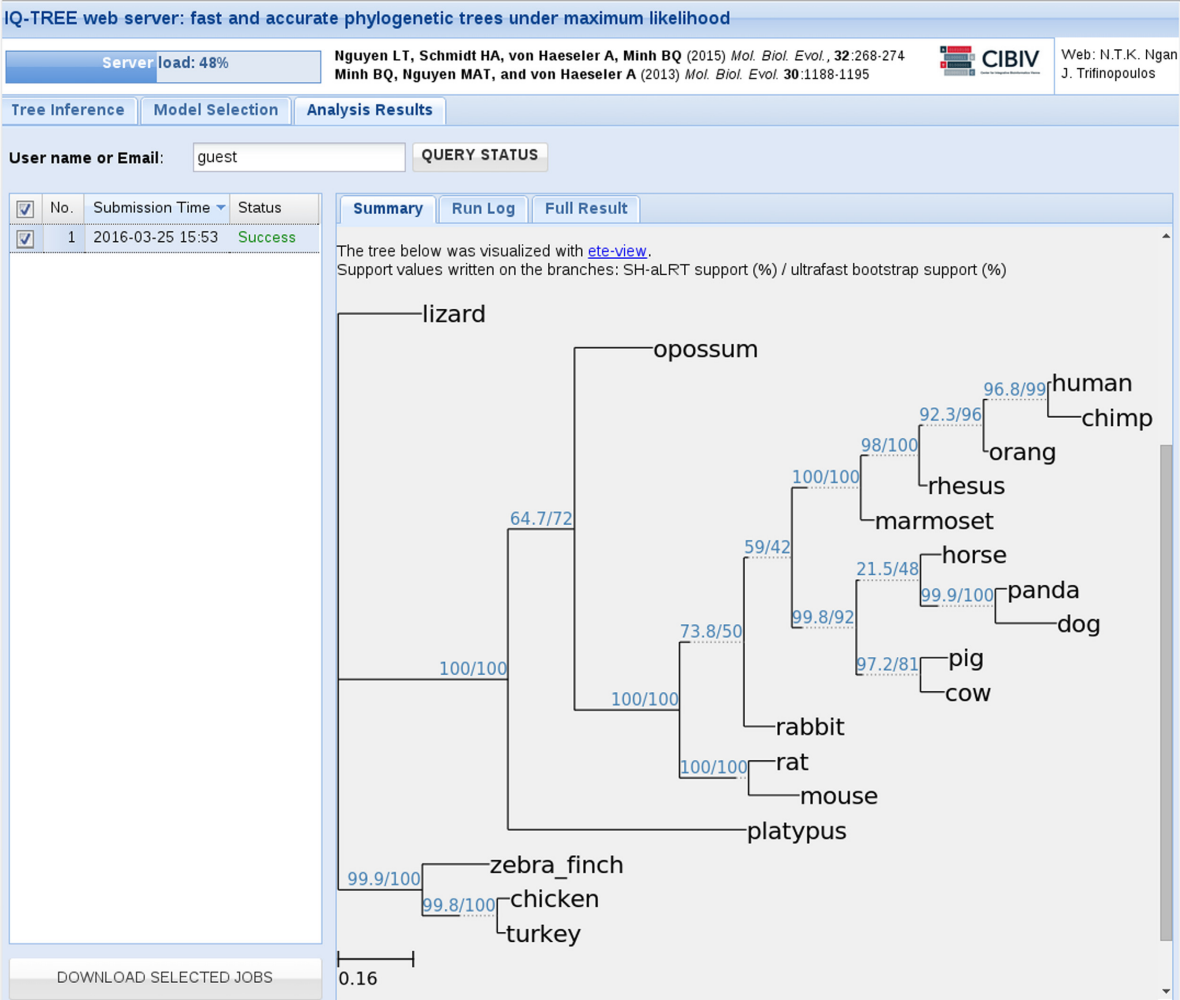
Screenshot of an example result with W-IQ-TREE for a chordate data set.

After job submission, W-IQ-TREE provides a URL that allows users to monitor the progress of the job(s). If an email address was provided, W-IQ-TREE automatically sends an email to inform the user that the job is done and where to access the results. Moreover, W-IQ-TREE will display the tree for a quick assessment of the result (Figure 2). The user can download the corresponding tree file in NEWICK, SVG and PDF formats for further analyses. Finally, a command line showing the user-specifications is provided to enable users to repeat the IQ-TREE run on a local computer system. Note that jobs requiring more than 24 CPU hours or >1GB RAM will be stopped if one of the limits is reached. In such cases, users are advised to download the checkpoint file and then resume a standard IQ-TREE run on local machines.

## AVAILABILITY

W-IQ-TREE is freely accessible at http://iqtree.cibiv.univie.ac.at. The W-IQ-TREE user interface was developed in Javascript using the Sencha framework (http://www.sencha.com), which works on most web browsers and platforms (e.g. Windows, Mac OSX and Linux). The server code was written in PHP to handle and distribute user jobs in the computing cluster. The source code of the W-IQ-TREE is available upon request. Tutorials and extensive documentation are available on the IQ-TREE homepage http://www.cibiv.at/software/iqtree/.
